# Comparative Lipidomics in Clinical Isolates of *Candida albicans* Reveal Crosstalk between Mitochondria, Cell Wall Integrity and Azole Resistance

**DOI:** 10.1371/journal.pone.0039812

**Published:** 2012-06-27

**Authors:** Ashutosh Singh, Vipin Yadav, Rajendra Prasad

**Affiliations:** Membrane Biology Laboratory, School of Life Sciences, Jawaharlal Nehru University, New Delhi, India; Université de Nice-CNRS, France

## Abstract

Prolonged usage of antifungal azoles which target enzymes involved in lipid biosynthesis invariably leads to the development of multi-drug resistance (MDR) in *Candida albicans*. We had earlier shown that membrane lipids and their fluidity are closely linked to the MDR phenomenon. In one of our recent studies involving comparative lipidomics between azole susceptible (AS) and azole resistant (AR) matched pair clinical isolates of *C. albicans*, we could not see consistent differences in the lipid profiles of AS and AR strains because they came from different patients and so in this study, we have used genetically related variant recovered from the same patient collected over a period of 2-years. During this time, the levels of fluconazole (FLC) resistance of the strain increased by over 200-fold. By comparing the lipid profiles of select isolates, we were able to observe gradual and statistically significant changes in several lipid classes, particularly in plasma membrane microdomain specific lipids such as mannosylinositolphosphorylceramides and ergosterol, and in a mitochondrial specific phosphoglyceride, phosphatidyl glycerol. Superimposed with these quantitative and qualitative changes in the lipid profiles, were simultaneous changes at the molecular lipid species levels which again coincided with the development of resistance to FLC. Reverse transcriptase-PCR of the key genes of the lipid metabolism validated lipidomic picture. Taken together, this study illustrates how the gradual corrective changes in *Candida* lipidome correspond to the development of FLC tolerance. Our study also shows a first instance of the mitochondrial membrane dysfunction and defective cell wall (CW) in clinical AR isolates of *C. albicans,* and provides evidence of a cross-talk between mitochondrial lipid homeostasis, CW integrity and azole tolerance.

## Introduction

The phenomenon of *Candida* cells acquiring multidrug resistance (MDR) is common. This however hampers their successful chemotherapy [1]–[4]. The *Candida* species infections by and large have been controlled by using the systemic azole drug fluconazole (FLC), and other topical azole and polyene drugs. The excessive use of FLC has however, resulted in the emergence of azole-resistant strains of *Candida* species [5]–[8]. *Candida albicans* as well as non-albicans species have evolved a variety of mechanisms to develop MDR to common antifungals. Reduced intracellular accumulation of drugs (due to rapid efflux) is one of the most prominent mechanisms of resistance in *Candida* cells. Accordingly, clinical azole resistant isolates of *C. albicans* display transcriptional activation of genes, encoding ATP Binding Cassette (ABC) multidrug transporter proteins CaCdr1p or CaCdr2p or Major Facilitator Super family (MFS) efflux pump protein CaMdr1p [5]–[8].

In *C. albicans*, lipids in addition to being the structural and metabolic components of yeast cells also play an important role in the frequently observed MDR. For example, CaCdr1p shows selectivity towards membrane recruitment and prefers membrane raft microdomains for its localization within plasma membrane [9]. There are close interactions between raft constituents such as ergosterol and sphingolipids (SLs), and disruption of these results in altered drug susceptibilities [10], [11]. Thus, any change in ergosterol composition by disruption of *ERG* genes, or change in SL composition by disruption of its biosynthetic genes, leads to improper surface localization of CaCdr1p [9]. Interestingly, MFS transporter CaMdr1p shows no such selectivity towards raft lipid constituents and remains fully membrane localized and functional in cells where SL or ergosterol biosynthesis is compromised [9]. There are also instances where common regulation of MDR and lipid metabolism genes have been observed [12], [13]. Changes in the status of membrane lipid phase (or membrane fluidity) and asymmetry also seem to affect azole resistance in *C. albicans* [14]. Taken together, MDR in *C. albicans* is closely linked to the membrane lipid composition. The overall drug susceptibility of a cell appears to be an interplay of membrane lipid environment, drug diffusion and extrusion [15]. Thus, it is quite apparent that lipids in some way or the other contribute to the development of resistant phenotype. However, the exact sets of changes that occur in the lipid composition leading to the resistant phenotype are not completely understood.

In our previous study, we had used a combination of high throughput mass spectrometry and statistical validation methods to analyze the lipidome of eight pairs of genetically matched clinical azole-susceptible (AS) and azole-resistant (AR) isolates of *C. albicans* [16]. We observed that although each AR isolate was similar with regard to displaying high MICs to drugs, each had a distinct lipid imprint. That study also revealed that each AR isolate was rather unique in terms of its lipidome, and that there were very few common lipid changes among AR isolates. The AS/AR matched isolates used therein were obtained from different patients (variable host environment); hence, the typical metabolic state of each AR isolate was not surprising [16]. In the present study, we have used a single clinical isolate of *C. albicans* recovered from an HIV-patient over a period of 2 years of FLC therapy during which time the level of FLC tolerance of the strain increased over 200-fold. Since these isolates were collected from a single patient, any possibility of variation in host environment and its effect on metabolic state of cells was minimized. We have subjected these isolates to high throughput MS-based platform and have performed detailed lipid profiling and compared the lipidomes of these sequential isolates of *C. albicans*. Our study provides a comprehensive picture of lipidomic changes which occur as the susceptible isolate develops tolerance to FLC. The spectrum of about 242 molecular species allowed us to identify and show how gradually their composition changed with increasing tolerance to FLC. The changes in ergosterol and SL-components of membrane microdomains, and in mitochondrial specific lipid phosphatidyl glycerol (PG) are most noteworthy. Our study shows an occurrence of a cross-talk between mitochondria, cell wall (CW) and *Candida* Drug Resistance (CDR) genes in the clinical scenario, in a sequential and related *C. albicans* isolate recovered from a single patient undergoing azole therapy.

## Results

### Shotgun screening of *Candida* Lipidome

The availability of genetically related variants recovered over a period of time from the same host enabled us to make a direct comparison of changes in lipidomes occurring in parallel to the development of resistance to FLC. These 17 isolates (TW1-17) which have been recovered by White's group have previously been characterized to show overexpression of CDR genes associated with the development of tolerance to FLC [17]. Of note, out of total isolates, we have chosen 6 isolates from 3 different time points of FLC therapy to cover the entire range of levels of FLC tolerance. Based on the MICs, our selection included: TW1 and TW2 which were highly susceptible to FLC (MIC_80_ ≤4µM), TW8 and TW9 had intermediate level of susceptibility to FLC (MIC_80_ ≤32µM) while TW16 and TW17 were highly tolerant to FLC (MIC_80_ >125µM). Before embarking upon lipidome analysis, we re-evaluated some of their phenotypic and molecular characteristics in these selected isolates which matched well with their published properties (Figure S1). For lipidome analysis, these selected isolates were harvested in the exponential growth phase and their total lipids were extracted as described in Materials and Methods [18]. The extracted lipids were subjected to ESI-MS/MS by direct infusion of the lipid extracts. The total amount of lipid was quantified as total normalized mass spectral signal of PGL (phosphoglycerides) + SL + SE (Sterol ester). The lipid content was found to range between 1018 to 1136 nmol per mg dry lipid weight in the *Candida* isolates analyzed (Sheet S1, worksheet 2).

Although, we determined lipids to their absolute amounts (as total normalized mass spectral signal of PGL + SL + SE), we have used the mole percentages (as % of total normalized mass spectral signal of PGL + SL + SE) for data analysis, which have lower standard deviation. By employing MS analysis, nine major PGLs classes which included phosphatidyl choline (PC), phosphatidyl ethanolamine (PE), phosphatidyl inositol (PI), phosphatidyl serine (PS), PG, phosphatidic acid (PA), lysoPC, lysoPE and lysoPG were targeted. Additionally, the lipid molecular species were identified by mass of the head group plus the mass of the intact lipid, allowing determination of the number of C atoms and double bonds in the acyl chain(s) of PGLs. The PGLs were quantified in relation to internal standards of the same lipid class. This procedure is known to provide accurate quantification because various molecular species of the same lipid class (here, the internal standard and other species) produce very similar amounts of mass spectral signal after electrospray ionization [19]. Our MS analysis also included four major groups of SLs, ceramides (CER), inositolphosphorylceramide (IPC), mannosylinositolphosphorylceramide (MIPC), mannosyldiinositolphosphorylceramide (M(IP)_2_C) and their relative amounts were determined. These observed lipid differences were also validated by evaluating select responsible genes of lipid metabolic pathways by reverse transcriptase PCR (RT-PCR) and by employing high performance – thin layer chromatography (HP-TLC). Initially, we did perform RT-PCR of some genes using all the isolates (Figure S1C) and found that the gene expression pattern was quite similar between isolate TW16 and TW17, and TW1 and TW2. Therefore for a direct comparison between most susceptible and tolerant isolate, RT-PCR was performed only with TW1 and TW17 isolates (discussed below).

### Most tolerant isolates have high levels of sterol esters

**Figure 1:**
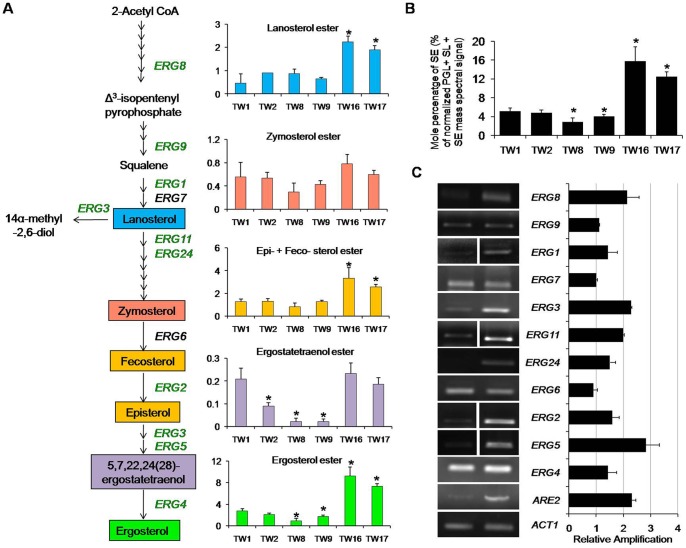
Upregulation of the sterol biosynthetic pathway in AR isolates. (A) Changes in the composition of major sterols and its intermediates among the sequential isolates of *C. albicans* were analyzed as described in methods. Green and black colour of gene in the pathway (A) represents upregulation and no change, respectively. (B) Total SEs are represented as % of the total PGL+ SE + SL mass spectral signal after normalization to internal standards and were determined as described in methods. (C) The fold change in gene expression levels of various *ERG* genes (relative amplification to *ACT1*) between TW1 (most susceptible to FLC) and TW17 (most resistant to FLC) were determined by RT-PCR. The gel picture is a representative of the RT-PCR analysis performed in replicates. Values in the histogram are means ± SD (n = 3 for all *Candida* strains). Asterisks “*” represents p<0.05. Lipid data taken from Sheet S1, worksheet 3 and 4.

Sterols were identified and quantified as SEs, as described in methods (Text S1). SEs ranged between 2.8 – 15.8% (% of total normalized mass spectral signal of PGL + SL + SE) which were accumulated up to 3 folds in TW16 and TW17, as compared to the less tolerant strain isolated at an earlier time point (Figure 1B). This accumulation of SEs in TW16 and TW17 was evident by a simultaneous increase in intermediate metabolites of sterol biosynthetic pathway and ergosterol ester, which was accumulated up to ∼5 fold (Figure 1A). While examining the FA composition of SEs, we found that all, C16:1, C16:0, C18:3, C18:2, C18:1 and C18:0 –FA containing SEs were elevated up to ∼4 fold in TW16 and TW17 as compared to the other FLC susceptible isolates (Sheet S1, worksheet 4). Using RT-PCR, we found that ergosterol biosynthetic pathway genes are upregulated in TW17 isolate as compared to susceptible TW1 (Figure 1C). An upregulated *ARE2* (an acyl CoA: sterol acyltransferase which regulates free sterol esterification) correlated well with the fact that most of the sterol predominantly accumulates as SEs in TW17 isolate (Figure 1C). Of note, we also noticed an unexplained difference between TW1 and TW2 with regards to the levels of *ERG11* expression (Figure S1C).

### MIPC content is depleted in resistant isolates

**Figure 2:**
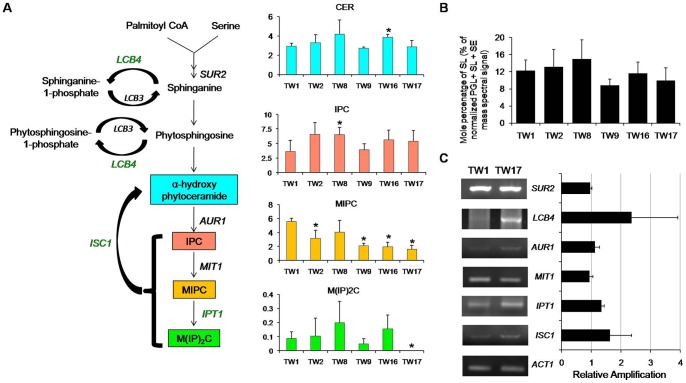
Downregulation of the SL biosynthetic pathway in AR isolates. (A) Changes in the composition of major SL classes among the sequential isolates of *C. albicans* were assessed as described in methods. Green and black colour of gene in the pathway represents upregulation and no change, respectively. (B) Total SLs are represented as % of the total PGL + SE + SL mass spectral signal after normalization to internal standards and were determined as described in methods. (C) The fold change in expression levels of various SL biosynthetic pathway genes (relative amplification to ACT1) between TW1 (most susceptible to FLC) and TW17 (most resistant to FLC) were determined by RT-PCR. The gel picture is a representative of the RT-PCR analysis performed in replicates. Values in the histogram are means ± SD (n = 3 for all *Candida* strains). Asterisks “*” represents p<0.05. Lipid data taken from Sheet S1, worksheet 4.

In *C. albicans*, it has been demonstrated that the interactions between membrane SLs and ergosterol are critical, and reduction in the content of either results in enhanced susceptibilities to drugs [9]–[11]. Therefore, to evaluate the impact of FLC exposure on SLs in TW isolates, we performed a detailed analysis as described in Text S1. We observed a trend of depletion in SL content in TW17 isolate in comparison to its susceptible counterpart TW1; however, this change was statistically not significant (Figure 2B). Using MS analysis, we further analyzed four major classes of SLs which included CER (detected as α-hydroxy phytoceramide), IPC, MIPC and M(IP)_2_C (Figure 2A). While the total SLs content did not vary significantly, MIPC was depleted up to 3.4 folds in TW17 as compared to its susceptible counterparts (Figure 2A). Of note, though IPC was accumulated by 1.5 fold in TW17 compared to TW1, but this difference was statistically not significant. M(IP)_2_C was below detection limits in TW17 isolate. We validated some of the genes of SL metabolism by semi-quantitative RT-PCR which revealed an overexpression of *LCB4* (D-erythro-sphingosine kinase) and *ISC1* (inositol phosphosphingolipidphospholipase C); however, statistically these changes were not significant (Figure 2C).

### Molecular species are responsive to the development of FLC tolerance

Based on the fatty acid (FA) composition and their positional distribution, each lipid class is sub-categorized into its molecular species [20]. A large number of these molecular lipid species and their numerous interactions with proteins and other lipids in yeasts provide flexibility in balancing their lipid composition in a variety of cellular processes [20]. Since majority of the major lipid classes were not significantly changed in FLC tolerant isolates, we evaluated that FLC exposure might lead to changes in the content and composition of different lipid molecular species. Therefore, we quantified the molecular lipid species by MS analysis of the extracts of TW isolates and detected molecular lipid species belonging to three major lipid groups (PGL, SL, and SE; see Sheet S1, worksheet 2). We could determine the abundance of over 242 species belonging to PGL, SL and SEs (Table S1 depicts changes only in the molecular species which were statistically significant) and observed that while adapting to the increasing FLC concentration, TW isolates show significant differences in the molecular species composition. For example, when we compared TW1 vs TW2, TW2 vs TW8, TW8 vs TW9, TW9 vs TW16 and TW16 vs TW17, the number of molecular species that undergo a significant change are about 60, 44, 28, 68 and 27, respectively. When we compared TW1 (most susceptible) with TW17 (most resistant), about 103 molecular lipid species of different lipid classes were found to be significantly variable (Sheet S1 (worksheet 3) and Table S1).

Notably, the changes in the molecular lipid species were gradual and correlated with the threshold of FLC tolerance among TW isolates. That each isolate has a typical molecular species profile was evident from principal component analysis (PCA) and hierarchal cluster analysis where these isolates could be separated as different clusters (Figure S2, Text S2 and Table S5). Moreover, this large scale lipid remodeling results in higher membrane order in FLC adapted strains as was evident from fluorescence polarization studies where TW17 has high *P*–value and low unsaturation index (both implying high membrane order) (Figure S3A and S3B).

### Long chain FA (LCFA) and odd chain FA levels respond to the development of FLC tolerance

A thorough examination of the molecular lipid species imprints of TW isolates revealed that there is accumulation of long chain FA containing ≥36-carbon PGL species, and depletion in ≤34-carbon containing PGL species with the increasing tolerance to FLC. This pattern was consistent among all PGL classes, and reflected FA chain lengths remodeling to maintain membrane composition in response to FLC stress (Table S2).

**Figure 3:**
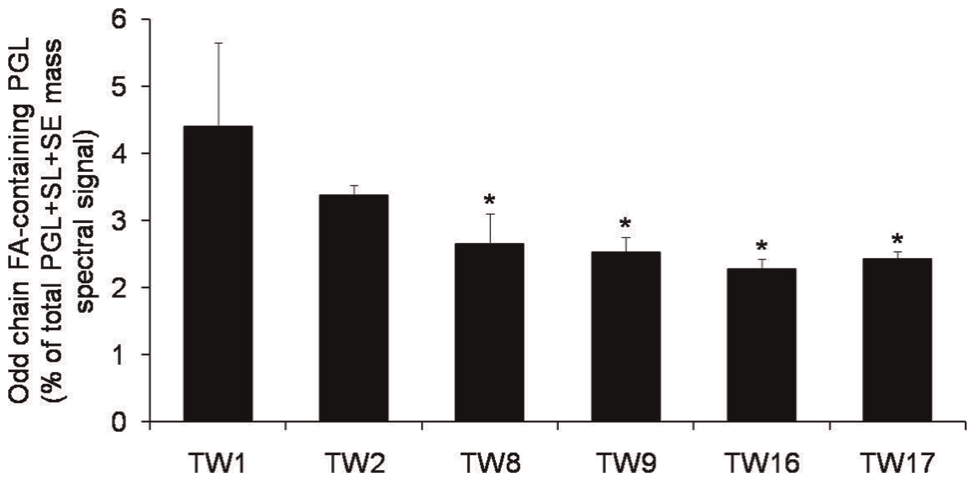
Accumulation of odd chain-FA containing PGLs in the FLC resistant isolates. Total amount of odd chain FA-containing PGL was calculated by adding the normalized amounts of each odd chain FA containing PGL molecular species (namely 31-C, 33-C, 35-C and 37-C containing PGLs). The data is represented as % of total PGL + SL + SE mass spectral signal after normalization to internal standards. Values are mean of 3 independent analyses (n = 3). Asterisks “*” represents p < 0.05. Lipid data taken from Sheet S1 (worksheet 3), Table S1 and Table S3.

We had earlier detected odd chain FA containing lipid species in various *Candida* species and in various AS/AR isolates to show that 31-, 33-, 35- and 37- carbon containing PGL species comprise the overall pool of the odd chain FAs in *Candida* [21]. Interestingly, we observed a depletion of odd chain FA containing PGL species with increasing tolerance to FLC exposure (Figure 3). These changes were more prominent in PC, PE, PI and PS species (Table S3). The large scale compositional changes in FAs of the PGL species are probably the result of the changes in the gene expression or changes in the levels of enzymes of FA metabolism (Table 1).

**Table 1:** Gene expression analysis of various genes between TW1 and TW17.

Category	Gene name	TW1	TW17	*p*-value
**PL metabolism**	*PSD1*	2.27±0.38	3.74±0.52	0.07
	*PSD2*	1.47±0.15	3.34±0.38	0.02
	*PEL1*	1.80±0.57	1.85±0.65	0.48
	*CDS1*	0.90±0.10	0.78±0.03	0.11
	*CRD1*	0.87±0.08	1.07±0.07	0.09
**Membrane pumps**	*CDR1*	1.45±0.09	2.43±0.16	0.02
	*CDR2*	1.43±0.01	2.44±0.07	0.00
	*MDR1*	2.26±0.11	2.82±0.01	0.02
**FA metabolism**	*FAS1*	0.70±0.02	0.73±0.03	0.26
	*FAS2*	4.68±0.01	5.89±0.08	0.00
	orf19.6443	1.09±0.02	0.94±0.04	0.03
	*EC11*	2.76±0.11	3.05±0.04	0.06
	*POT1-2*	6.83±0.18	20.6±1.00	0.00
	orf19.3859	0.99±0.07	1.53±0.18	0.05

Transcript levels were determined by RT-PCR as described in Methods. The data represents relative amplification to *ACT1*. Values are means ± SD (n = 3) along with the *p*-values.

### Mitochondrial PG levels are lower in resistant isolates

**Figure 4:**
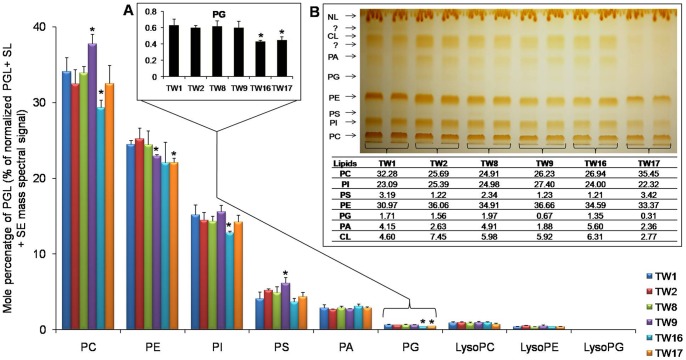
The sequential isolates of *C. albicans* shows modulation of PGL metabolism in the AR isolates. Changes in the composition of major SL classes among the sequential isolates of *C. albicans* were assessed as described in methods. Data is represented as % of the total PGL+ SE + SL mass spectral signal after normalization to internal standards. Inset A depicts in larger scale, the change in PG levels among the sequential isolates of *C. albicans*. Values are mean of 3 independent analyses (n = 3). Data taken from Sheet S1, worksheet 4. Inset B shows PL analysis of strains by HP-TLC: cells were grown overnight (to the exponential growth phase) in YEPD medium. Lipids were extracted as described earlier [18] and separated by thin-layer chromatography using chloroform: methanol: acetic acid (65:28:8) as the solvent system. The mobility of CL, PA, PG, PE, PS, PI and PC are indicated after iodine staining (n = 2). The corresponding values of each lipid class as mol % are also indicated.

Our method could detect six major PGLs in the abundance order PC>PE>PI>PS>PA>PG, which did not change between the AS and AR pairs (Figure 4). PC, PE and PI accounted for almost 80% PGLs in all the isolates. As shown in figure 4 (Inset A), contents of mitochondrial lipid PG decreased by as much as 1.4 fold in TW17 as compared to most susceptible TW1. However, MS based lipidome analysis and HP-TLC revealed that there was no statistically significant change in most of the other PGLs (Figure 4). PG is metabolic precursor of cardiolipin (CL) which is a major mitochondrial anionic phospholipid (PL) with important functions in promoting cell growth, anaerobic metabolism and mitochondrial biogenesis [22]. We observed that a depletion of PG levels was also associated with a simultaneous decrease in CL levels (Figure 4, inset B). PGLs composition was also validated by HP-TLC which showed a drop in PG and CL in most FLC tolerant TW17 isolate (Figure 4, inset B). It should be noted that we could only measure CL content by HP-TLC.

However, the RT-PCR data did not show any significant changes in the transcript levels of *CDS1* (a CDP-diglyceride synthetase) *PEL1* (a phosphatidylglycerolphosphate synthase) and *CRD1* (CL synthase) between TW1 and TW17 isolates (Table 1). Of note, we observed an apparent gradual decrease in CL levels with increasing FLC tolerance, but the overall ratio of CL/PG was maintained in most of the isolates. For example, in TW17 the CL is decreased by ∼1.6 fold but the ratio of CL/PG is similar to TW16 where the contents of CL are rather higher, compared to TW1 isolate. Apparently, the required CL levels necessary for proper functioning of mitochondria are largely maintained and it is the anionic-PG, whose imbalanced level most likely affects the mitochondrial membrane (discussed below).

### Decreased PG level leads to compromised mitochondrial membrane and CW integrity

**Figure 5:**
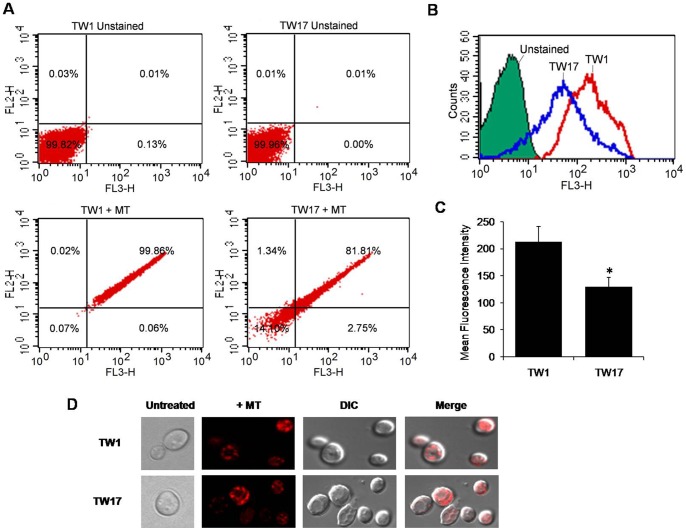
Measurement of mitochondrial membrane dysfunction among TW1 and TW17 isolates. For the flow cytometry analysis, cells were stained with 100 nM MitoTracker Red CMXRos (which fluoresces upon oxidation in respiring mitochondria). (A) Quadrant plots, (B) histograms and (C) bar graph of flow cytometry analysis between TW1 and TW17 are depicted (n = 4). (D) The red fluorescence of MitoTracker Red CMXRos was also visualized by confocal microscopy.

We evaluated if reduction in the mitochondrial specific lipid PG would affect mitochondrial integrity. For this, we used cell-permeant mitochondrion-selective dye Mito Tracker Red CMxROS which is a good indicator of mitochondrial integrity [23], [24]. Interestingly, compared to TW1, we observed ∼40% decrease in mean fluorescent intensity in binding of the probe in TW17 isolate (Figure 5A, 5B and 5C). Of note, no visual difference in the mitochondrial integrity could be deduced by confocal microscopy of labeled TW1 and TW17 isolates (Figure 5D). Moreover, the TW17 did neither show much alteration in mitochondrial respiratory activity as was deduced from their ability to grow normally on non-fermentable carbon sources or at elevated temperatures (data not shown), nor did the TW17 show any defects in ROS generation as indicated by flow cytometry and confocal microscopy using DC-FDA (molecular probes) (data not shown).

**Figure 6:**
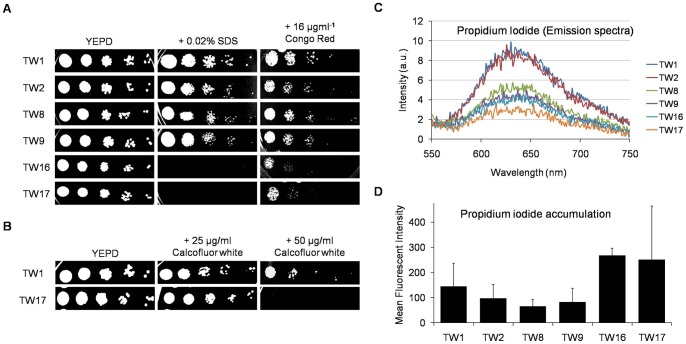
FLC exposure alters CW integrity in the sequential isolates of C. albicans. (A) Sequential isolates were tested with CW perturbing agents like Triton X-100 (upto 0.04%), 0.02% SDS, 16 µgml^−1^ Congo Red. (B) Susceptibility to Calcofluor White (upto 50 µgml^−1^) was tested. These spot tests were performed as described in methods. (C) Passive diffusion rate of PI was monitored by spectrofluorimeter. Briefly, the cells were pre-incubated with 3 µM PI for 45 min and then centrifuged. The supernatant was taken and the emission spectrum of PI was recorded for each strain. (D) The cells with PI were subjected to flow cytometry analysis to measure the amount of PI accumulated in each strain. The data is represented as the mean fluorescent intensity (n = 2).

The CW integrity is linked to mitochondria and PL homeostasis [25], however, the link between the mitochondria, CW integrity and azole resistance is largely unexplored in *C. albicans*. Interestingly, we observed that TW17 isolate was susceptible to the CW perturbing agents SDS, Congo red and Calcofluor white (Figure 6A and 6B). The compromised CW integrity of TW17 isolate could further be confirmed by increased passive diffusion and accumulation of otherwise impermeate fluorescent PI (Figure 6C and 6D).

**Figure 7:**
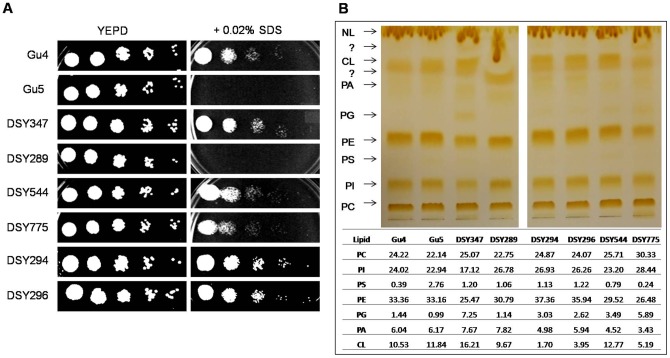
FLC exposure leads to gradual development of a partially compromised CW and affects PG biosynthesis in several other clinical isolates of *C. albicans*. A. Several pairs of isogenic clinical isolates of *C. albicans* were tested with 0.02% SDS, a CW perturbing agent. The spot tests were performed as described in methods. B. PL analysis of various AS/AR clinically matched isogenic isolates by HP-TLC. Briefly, cells were grown overnight (to the exponential growth phase) in YEPD medium. Lipids were extracted as described earlier [18] and separated by thin-layer chromatography using chloroform: methanol: acetic acid (65:28:8) as the solvent system. HP-TLC analysis of each match pair and the corresponding values of each lipid class as mol % are also indicated.

A correlation with PG content with CW integrity and mitochondrial functions was further evident from our other data. We used two other matched pair clinical isolates of *C. albicans*, Gu4/Gu5 and DSY347/DSY289 (where Gu5 and DSY289 are resistant to drugs). Both Gu5 and DSY289 had lowered PG levels (∼1.5 and ∼6.4 fold, respectively) as well as compromised CW as compared to their susceptible counterparts (Figure 7A and B).

In another matched pair DSY544/DSY775, where the PG content was higher in resistant DSY775 as compared to its sensitive counterpart DSY544 (Figure 7B), no CW defect was evident (Figure 7A). In the DSY294/DSY296 isogenic pair where PG content did not change between sensitive DSY294 and resistant DSY296 (Figure 7B) also had no evident CW damage (Figure 7A). Notably, we could not see consistent differences in the lipid profiles of these AS and AR pairs (CL levels), probably because they come from different patients, however, a correlation with PG content with CW integrity and mitochondrial functions was further evident from our other data.

## Discussion

Prolonged exposure of C. albicans to FLC allows cells to develop tolerance to higher concentrations of the drug [5]–[8]. Hence, AR cells display considerably decreased susceptibility to the drugs (high MICs). An overexpression of efflux pump proteins like Cdr1, Cdr2, and Mdr1 is one of the main contributors of azole resistance, which necessitates several compensatory adaptive changes, that includes alterations in lipid metabolism of *Candida* [5]–[8], [12], [13], [17]. The importance of lipids in the physiology of *Candida* including in MDR is well established [9]–[11], [14]–[16]. However, exactly how lipids adapt or change, are some of the aspects which are not well understood.

Previously, in an attempt to evaluate the impact of FLC on the lipid regulatory circuitry behind MDR in *C. albicans*, we had performed a comparative lipidomics of AS and AR clinical isolates of *C. albicans* isolated from different patients [16]. In the present study, to minimize any variations due to host/pathogen niches, we have used sequential isolates earlier isolated by Ted White's group, from a single HIV-infected patient who was on FLC therapy for over a period of 2 years [17]. In this TW series of 17 isolates (TW1-17), each isolate represents an isogenic precursor parent of the next isolate. A comparative snap shot lipidomic status of selected sequential AR isolates of *C. albicans* gave interesting insights.

### Membrane raft specific lipids are selectively affected in AR isolates

In *C. albicans*, ergosterol which is predominantly localized within microdomains “lipid raft”, is involved in many physiological functions and provides surface localization platform for membrane proteins [9]–[11], [26], [27]. Thus, the observed raised levels of ergosterol in highly resistant TW16 and 17 (validated by RT-PCR) (Figure 1) could be linked to an overexpressed level of Cdr1 protein, since it is preferentially localized within the lipid raft microdomains [9]. Recently, Zhang *et* *al*. demonstrated that ergosterol levels are required in V-ATPase function which forms the cellular basis of azole toxicity in *C. albicans* [28]. Further, the mammalian presynaptic serotonin transporter (SERT) is functionally overexpressed only in the presence of cholesterol [29].

In contrast to ergosterol, there was a marked depletion of another major constituent of membrane microdomains. One of the complex SL members, MIPC contents was significantly lower in TW16/17 isolate as compared to TW1 (Figure 2A). This depletion in MIPC content may appear odd for maintenance of the raft domain. However, there are instances to show that a minimum level of SL is sufficient to maintain the raft integrity and efflux function [30]. Therefore, it could be argued that observed depletion of SLs which is probably the result of azoles induced compensatory changes in TW17 remains within its critical limits and along with the raised ergosterol, continues to provide optimal platform to maintain the functionality of overproduced Cdr1 protein.

### Azole resistance necessitates remodeling of molecular species of lipids

In yeasts, although structural classes of lipids are limited, they have the potential to generate up to several thousand different molecular species to bring in diversity in lipid composition [20]. Our present results clearly demonstrate that exposure of *C. albicans* to FLC triggers massive remodeling of molecular lipid species in TW isolates (Table S1, Sheet S1, worksheet 2 and 3). The fact that ∼103 molecular lipid species were found to be significantly variable between TW1 and TW17 implies that there is large scale remodeling of molecular lipid species while the isolate gradually adapts to FLC toxicity. Since the intracellular drug concentration is dependent not only on active drug efflux, but also on the rate of its import, the order of the membrane lipid bilayer core becomes an important determinant in the development of drug resistance [14], [15], [26].

### AR isolates display an increase in LCFA and depletion in odd chain FAs

The LCFA's are functionally very relevant in bacterial models but in yeasts like *C. albicans* these are poorly understood [21], [31], [32]. In *C. albicans*, it is quite understandable that the membrane PLs might incorporate FAs of varying length and saturation to maintain the optimal membrane order [33]. Precisely, we observed an accumulation of LCFA containing ≥36-carbon PGL species at an expense of ≤34-carbon containing PGL species, with the increasing FLC tolerance (Table S2). Since the FAs with lesser chain lengths, and higher unsaturation are less rigid [34], therefore an increased pool of LCFA (of ≥36-carbon containing PGLs) might directly be contributing to maintain the high order of the membrane.

Notably, we also observed a progressive depletion of typical odd chain FA containing PGLs with the increasing tolerance to FLC (Figure 3 and Table S3). Although this study for the first time points towards odd chain FA containing PGLs role in drug tolerance adapting mechanisms of pathogenic *C. albicans*, a complete understanding of the physiological relevance of it requires further validation. If explored in detail, possibly like in animal models of environmental and food web studies, these odd chain FAs could serve as useful biomarkers for *C. albicans* drug resistance [35]–[37].

Together, changes like increased pool of LCFA's and depletion of odd-chain FAs along with variations in molecular lipid species are the reflection of close knit maintenance of membrane lipid homeostasis. Of note, the fact that both odd chain FAs and very-LCFA (generally up to 22 carbons) are well established features of plant lipids [36], [38], the presence of these lipids in human pathogenic *C. albicans* is of great evolutionary significance [21].

### A change in PG and CL in AR isolates points a link between mitochondria and CW integrity

Recently, mitochondria have been identified as an important contributor to virulence and drug tolerance of human fungal pathogens [39]–[43]. For example, mitochondrial mutants of yeast display altered drug susceptibility to drugs, particularly those which target cell membranes, such as the azoles and the polyenes [25], [41], [43], [44]–[47]. Apparently mitochondrial function in membrane lipid homeostasis is the common denominator underlying the observed effects of mitochondria in drug tolerance and CW integrity. The mechanistic details, particularly how these two pathways are linked, are far from understood. The observed changes in the levels of mitochondrial lipid PG led us to speculate if mitochondrial function is compromised in these isolates. Notably, a decrease accessibility of mito-tracker dye to TW17 cells as compared to TW1 cells provided sufficient evidence of compromised mitochondria (Figure 5); however, apparently the damage was restricted to mitochondrial membrane structure since it was not enough to cause respiratory defect and the accumulation of ROS (data not shown). This also supports an earlier observation that respiratory deficiency *per se* is not sufficient for eliciting MDR in yeast [43], [47].

There are examples to suggest a possible link between mitochondrial dysfunction, lipid homeostasis and CW integrity [25], [48]. Reportedly, *S. cerevisiae* and *C. glabrata* mitochondrial mutants most susceptible to caspofungin were those with impaired mitochondrial biosynthesis of the PLs namely PG and CL [25], [43], [49]–[51]. We observed that AR isolate TW17 that had lower levels of PG also possessed damaged CW as was evident from its sensitivity to CW disrupting agents and TEM images. Notably, we could also indirectly correlate CW damage and azole resistance with mitochondrial PG content by assessing CW damage in another set of matched pairs of AS and AR isolates. We observed that AR isolates which had lower PG content were also susceptible to CW disrupting agents as compared to those pairs which were resistant to azoles but showed no change in PG levels. We suspect that it is PG levels rather than CL that are more linked to mitochondrial defect and CW integrity. This notion is supported by a recent study, where it is demonstrated that nulls of *PEL1* (encodes PG biosynthetic gene) in *C. albicans* have damaged CW and decreased susceptibility to FLC [52]. Notably, CL defects in mitochondrial membranes can easily be compensated for by PE, with the two PLs having overlapping roles [53]–[55]. Our MS and HP-TLC did not reveal any significant change in PE levels between these isolates (Figure 4). However, this was not reflected from our RT-PCR data analysis where both *PSD1* and *PSD2* (the phosphatidylserine decarboxylases that catalyze the conversion of PS to PE) were upregulated in TW17 isolate as compared with its susceptible counterpart (Table 1).

Notably, in certain matched clinical isolates (DSY775/DSY296) neither PG levels nor any defect in CW integrity was evident and yet these isolates displayed reduce susceptibility to FLC. It emphasizes the fact that that the development of MDR in *C. albicans* employs multiple pathways [5]–[8].

Although our data establishes a link between altered lipid homeostasis, mitochondrial dysfunction and CW integrity in the development of azole tolerance in *C. albicans*, how these events lead to the activation of MDR pathway is not clear. It has been shown that mitochondrial dysfunction can lead to the activation of Pdr pathway in *S. cerevisiae* [56]. The available evidence in *S. cerevisiae* points to a link between mitochondrial control over membrane structure and lipid homeostasis and the role of the Pdr pathway in membrane biogenesis [43], [57]–[59]. For example, deletion of *ScOXA1* (a mitochondrial inner membrane insertase with cytochrome oxidase activity) results in increase drug resistance without any apparent loss in mtDNA [47], [59]. Loss of *PGS1* (a phosphatidylglycerolphosphate synthase that catalyzes the synthesis of phosphatidylglycerolphosphate from CDP-diacylglycerol and sn-glycerol 3-phosphate) also results in decrease susceptibility to drugs independent of mtDNA loss [39], [57]–[61]. It is shown that in Psd1 in respiratory deficient yeast is partly dissociated and results in activation of MDR pathway [43], [62]. However, one needs to uncover regulatory pathway (s) interlinking CDR genes with mitochondrial functions in *C. albicans*.

Although it is not yet clear how and in which order these events are interlinked and triggered, but one could safely conclude that an extensive membrane remodeling and cellular lipid signaling is essential for the survival of azole stress and for the up-regulation of genes coding for multidrug transporters. Several recent studies have highlighted the importance of mitochondrial function for tolerance of antifungal drugs but the exact molecular mechanisms are not fully understood. Our study provides first clinical evidence wherein an interplay between mitochondria, CW and lipid homeostasis in the development of azole tolerance is evident.

## Materials and Methods

### Strains, media and culture conditions

*C. albicans* strains used in this study are listed in Table S4 [16], [17], [67], [68] and will be described as TW series of isolates throughout the study. *C. albicans* cells were kept on YPD plates and used to inoculate in YPD medium (1% yeast extract, 2% glucose, and 2% bactopeptone). For solid media, 2.5% agar was added. The cells were diluted into 50 ml fresh medium at 0.1 OD at A_600_ (∼10^6^ cells/ml) and grown until the cells reached the density of ∼2×10^8^ cells/ml at 30°C (approximately in 14 hrs). Three separate cultures of each *Candida* isolate were used.

### Lipid standards

Synthetic lipids with FA compositions that are not found, or are of very low abundance in *Candida,* were used as internal standards. Lipid standards were obtained from Avanti Polar Lipids (Alabaster, AL).

### Lipid Extraction

Lipids were extracted from *Candida* cells using a slight modification of the method of Bligh and Dyer [18]. Briefly, the *Candida* cells were harvested at exponential phase and were suspended in 10 ml methanol. 4 g glass beads (Glaperlon 0.40–0.60 mm) were added and the suspension was shaken in a cell disintegrator (B. Braun, Melsungen, Germany) four times for 30 sec with a gap of 30 sec between shakings. Approximately 20 ml chloroform was added to the suspension to give a ratio of 2∶1 of chloroform: methanol (v/v). The suspension was stirred on a flat-bed stirrer at room temperature for 2 hrs and then filtered through Whatman No. 1 filter paper. The extract was then transferred to a separating funnel and washed with 0.2 volumes of 0.9% NaCl to remove the non-lipid contaminants. The aqueous layer was aspirated and the solvent of the lipid-containing, lower organic layer was evaporated under N_2_. The lipids were stored at −80°C until they were analyzed.

### ESI-MS/MS lipid profiling

An automated ESI-MS/MS approach was used to quantify PGL, SL and SE. Data acquisition and analysis was carried out as described previously by Devaiah *et* *al*. and Singh *et* *al*. [16], [26], [63]. Precise amounts of internal standards, obtained and quantified as previously described by Welti *et* *al*. and Singh *et* *al*. [16], [26], [64], were added. Processing of the data, including isotope deconvolution, was done similar to the way described by Singh *et* *al*. [16], [21]. For details, see Text S1.

### High Performance – Thin Layer Chromatography (HP-TLC)

The *Candida* cells were grown overnight (to the exponential growth phase) in YEPD medium. Lipids were extracted as described earlier [18]. Following extraction samples were processed on the automated HP-TLC system (CAMAG, Muttenz, Switzerland) according to the instructions of the manufacturer. Ready-to-use silica coated plates (Merck) were activated in 80°C for 30–45 min. Sample volume equivalent to 120µg lipid was applied in band-form using specialized Hamilton syringe on one-side of the TLC plate in individual tracks. The TLC plate was developed in chloroform: methanol: acetic acid (65∶28∶8) solvent system. The plate was developed in the automated developing chamber (CAMAG) until the solvent front reached the maximum distance (80 mm distance in a typical 20×10 cm plate). The developed plate was dried and separated lipids were stained with iodine for visualization of various PLs. Then subsequent charring with 470 mM CuSO4 (in 8.5% *o*-phosphoric acid) was carried out. The individual Rf values of peaks along with their peak areas were obtained. These data were matched with the lipid standards.

### Drug susceptibility

Drug susceptibilities were measured by using microtiter plate and spot assays. The MICs for the strains were determined with a broth microdilution method as described previously [15]. For the spot assay, 5-μl samples of fivefold serial dilutions of each yeast culture (each with cells suspended in normal saline to an optical density at 600 nm [OD600] of 0.1) were spotted onto YEPD plates in the absence (control) or in the presence of various concentrations of FLC. Growth differences were recorded following incubation of the plates for 48 h at 30°C.

### Mitochondrial integrity

The integrity of mitochondria was determined using MitoTracker Red CMXRos (Molecular probes, Invitrogen). Briefly, cells (equal to 0.1 O.D) were stained with 100 nM MitoTracker Red CMXRos (which fluoresces upon oxidation in respiring mitochondria) at 30°C for 45 min. Flow cytometry of the stained cells was performed as described previously [23]. The red fluorescence of MitoTracker Red CMXRos was also visualized by confocal microscopy as described previously [23], [24].

### CW susceptibility

Spot tests were performed (as described above) on YEPD plates in the absence (control) or in the presence of the following agents: SDS (0.02%), congo red (16 μg/ml) and calcofluor white (upto 50 μg/ml). The plates then incubated for 48 hrs at 30°C, recorded growth differences.

### Membrane permeabilization assays

The permeability changes in *Candida* isolates was monitored by the impermeant dye PI (Propiduim Iodide). For this, *C. albicans* cells (∼1×10^6^) were first harvested at the logarithmic phase and suspended in PBS (phosphate buffer saline). Cells were incubated in water bath set at 30°C at 200 rpm with 3.0 μM (PI) for 45 min. After incubation, cells were harvested by centrifugation. The aliquots of the cell suspension were taken in separate vials and then cells were re-suspended in PBS [65]. Flow cytometry was performed with a Calibur flow cytometer (Becton–Dickinson, San Jose, CA, USA). Further, the aliquots after PI treatments (those collected in flow cytometry experiment) were taken and the emission spectrum of PI was recorded for each strain using a fluorescence spectrophotometer (CARY-Eclipse, Varian Inc., Netherlands). The excitation and emission wavelengths used for PI were 536 and 617 nm respectively.

### Reverse transcriptase – polymerase chain reaction (RT-PCR)

RNA isolation was done using RNeasy Mini Kit (QIAGEN Inc., USA) as per manufacturer's specifications, except that 300μl acid washed 0.4–0.6 mm glass beads (Sigma, St. Louis, MO) were used during cell lysis. RNA was precipitated using absolute ethanol and washed twice with 80% ethanol, dried and resuspended in 50 μl DEPC treated water at 58^o^C. RNA obtained was quantitated spectrophometrically and it was also electrophoresed in denaturing formaldehyde gel.

RT-PCR was done using the RevertAid^TM^ H Minus kit (MBI, Fermentas). Briefly, 1µg isolated RNA was primed with oligo (dT)_18_ for cDNA syntheis at 42°C for 60 min. Reverse transcription reaction was terminated by heating at 70°C for 5 min. The synthesized cDNA product (2µl) was directly used for PCR amplification reaction (50 µl) using gene specific forward and reverse primers. The amplified products were subjected to gel electrophoresis and were quantitated by using Quantity One software with the Bio-Rad gel documentation system. The densities of bands (for genes of interest) were measured and normalized to that of the endogenous gene. The oligonucleotide sequences used for RT-PCR are listed in Sheet S2. Of note, our RT-PCR analysis included most of the responsive genes of lipid metabolism as was earlier detected from the microarray analysis of these isolates [66].

### Statistical Analysis

The mean of three independent biological replicates ± standard deviation (SD) from the individual samples was used to compare the lipids of *Candida* strains. Multivariate data analysis (pattern recognition) was employed. PCA was performed using the software SYSTAT, version 10 (Systat Software Inc., Richmond, CA, USA) using three replicates of each of the AS and AR *Candida* isolate to highlight the statistically significant lipid differences. To assess the statistical significance of the difference in lipid datasets and PC scores, the Student *t*-test was performed using the significance level of 0.05. Hierarchical cluster analysis was also performed using SYSTAT, version 10. When all the values for a particular lipid species were zero in all samples, the data for that lipid species were removed from the analysis. The data in percentage were log-transformed and normalized to the same scale for PCA and hierarchical cluster analysis.

## Supporting Information

Figure S1 **Phenotypic and molecular properties of the sequential isolates of** ***C. albicans*** **used in this study.** Isolates are shown on the y axis in the order in which they were obtained from the patient. MICs of FLC were determined by (A) liquid FLC susceptibility assay and (B) the spot assay, as described in Methods. Approximate numbers of cells (per ml) are indicated at the top of panel B. MICs are reported as MIC_80_ (µM). (C) Genetic changes were identified as the gene expression levels of *CDR1*, *MDR1* and *ERG* genes as determined by RT-PCR, described in Methods. Values shown as MICs are mean of 3 independent analyses. Spots and RT-PCR result was repeatable at least in 2 independent analyses.
(TIF)

Figure S2 **Principal component analysis (PCA) and hierarchical cluster analysis of lipid species amongst the sequential isolates of** ***C. albicans*** **.** PCA was performed using the software SYSTAT, version 10 as described in Text S2. The scores for the first three principal components, explaining 53% of the variance, were plotted. Each point in the plot is the mean of corresponding replicate's principal component scores. (A) The scores plot of principal component 1 (27.4% of variance) vs principal component 2 (15.2%). (B) The scores plot of principal component 1 (27.4%) vs principal component 3 (10.8% of variance). (C) Hierarchical cluster dendrogram was prepared using lipidome profiles of the TW isolates. Hierarchical clustering was performed using the software SYSTAT, version 10 as described in Text S2.
(TIF)

Figure S3 **Membrane fluidity and unsaturation index measurements in the sequential isolates of** ***C. albicans*** **.** (A) Membrane fluidity was assessed in terms of fluorescence polarization measurements, which were carried out using fluorescent probe 1,6-diphenyl-1,3,5-hexatriene (DPH), as a reporter. Brieﬂy, cells were incubated with Zymolyase (100 U/g wet weight) at 37°C for 3 h with gentle shaking to remove the CW. Spheroplast preparation was monitored turbidometrically by checking the ability of 0.2% sodium dodecyl sulfate to lyse the enzyme-digested cells. Fluorescence polarization was measured at excitation and emission wavelengths of 360 and 426 nm, respectively. The measured ﬂuorescence intensities were corrected for background ﬂuorescence and the light scattering from the unlabeled sample. (B) Degree of unsaturation was determined by calculating the unsaturation index (UI) of the PGLs. UI was calculated as follows: UI  =  [(1 x % monoene-PGL) + (1 x % diene-PGL) + (1 x % triene-PGL) + (1 x % triene-PGL) + (1 x % tetraene-PGL) + (1 x % pentaene-PGL) + (1 x % hexaene-PGL)]/100. Values are mean of 3 independent analyses (n = 3). Asterisks “*” represents p<0.05. Lipid data taken from Sheet S1, worksheet 3.
(TIF)

Sheet S1 **Lipid amounts of lipid classes and molecular species.** Worksheet 1 is the information sheet. Worksheet 2 has the absolute amounts (nmol per mg dry lipid wt.) of the molecular lipid species. Worksheet 3 has the mol percentages (% total mass spectral signal of PGL + SL + SE) of the molecular lipid species. Worksheet 4 the total amounts of various lipid classes as the mol percentages (% total mass spectral signal of PGL + SL + SE).
(XLS)

Sheet S2 **Oligonucleotides used in this study for RT-PCR.**
(XLS)

Table S1 **Changes in molecular lipid species upon FLC stress. Blue and Red colours represent decreasing and increasing trends respectively.** The length of the colour in each cell represents the predominance of a lipid species. A total of 242 lipid species were detected, however, table depicts only those lipid species which showed differences between TW1 and TW17 with p-values <0.05. Values are represented as % of the total PGL + SE + SL mass spectral signal and the data taken from Sheet S1, worksheet 3.
(DOC)

Table S2 **Abundance of PGLs based on their total number of carbons in the FA chains among various isolates used in this study.** The data is represented as % of total PGL + SL + SE mass spectral signal after normalization to internal standards. Values are mean of 3 independent analyses (n = 3). Asterisks “*” represents p<0.05. Data taken from Sheet S1, worksheet 3.
(DOC)

Table S3 **Changes in the contents of odd chain FA –containing PGL classes among various isolates used in this study.** Total amount of odd chain FA-containing PGLs for each lipid class was calculated by adding the normalized amounts of each odd chain FA -containing PGL molecular species of that particular class (namely 31-C, 33-C, 35-C and 37-C containing PGLs). Odd chain FA -containing lipid species were not detected in PA class. The data is represented as % of total PGL + SL + SE mass spectral signal after normalization to internal standards. Values are mean of 3 independent analyses (n = 3). Asterisks “*” represents p<0.05. Data taken from Sheet S1, worksheet 3.
(DOC)

Table S4 **Strains used in the study.**
(DOC)

Table S5 **Loadings of principal components 1, 2 and 3.** The 12 highest and 12 lowest values are indicated. In red colour are shown the molecular lipids species that match with our previous prediction of molecular species that are more responsive during the azole stress [16].
(DOC)

Text S1 **ESI-MS/MS lipid profiling.**
(DOC)

Text S2 **Principal Component Analysis (PCA) and Hierarchal cluster analysis of lipid species quantitative data distinctly separates susceptible and resistant isolates.**
(DOC)
