# Supplementary figures and images for: Comparative Lipidomics in Clinical Isolates of Candida albicans Reveal Crosstalk between Mitochondria, Cell Wall Integrity and Azole Resistance
Source: PLoS One. 2012 Jun 27;7(6):e39812. doi: 10.1371/journal.pone.0039812 (PMC3384591; doi:10.1371/journal.pone.0039812)

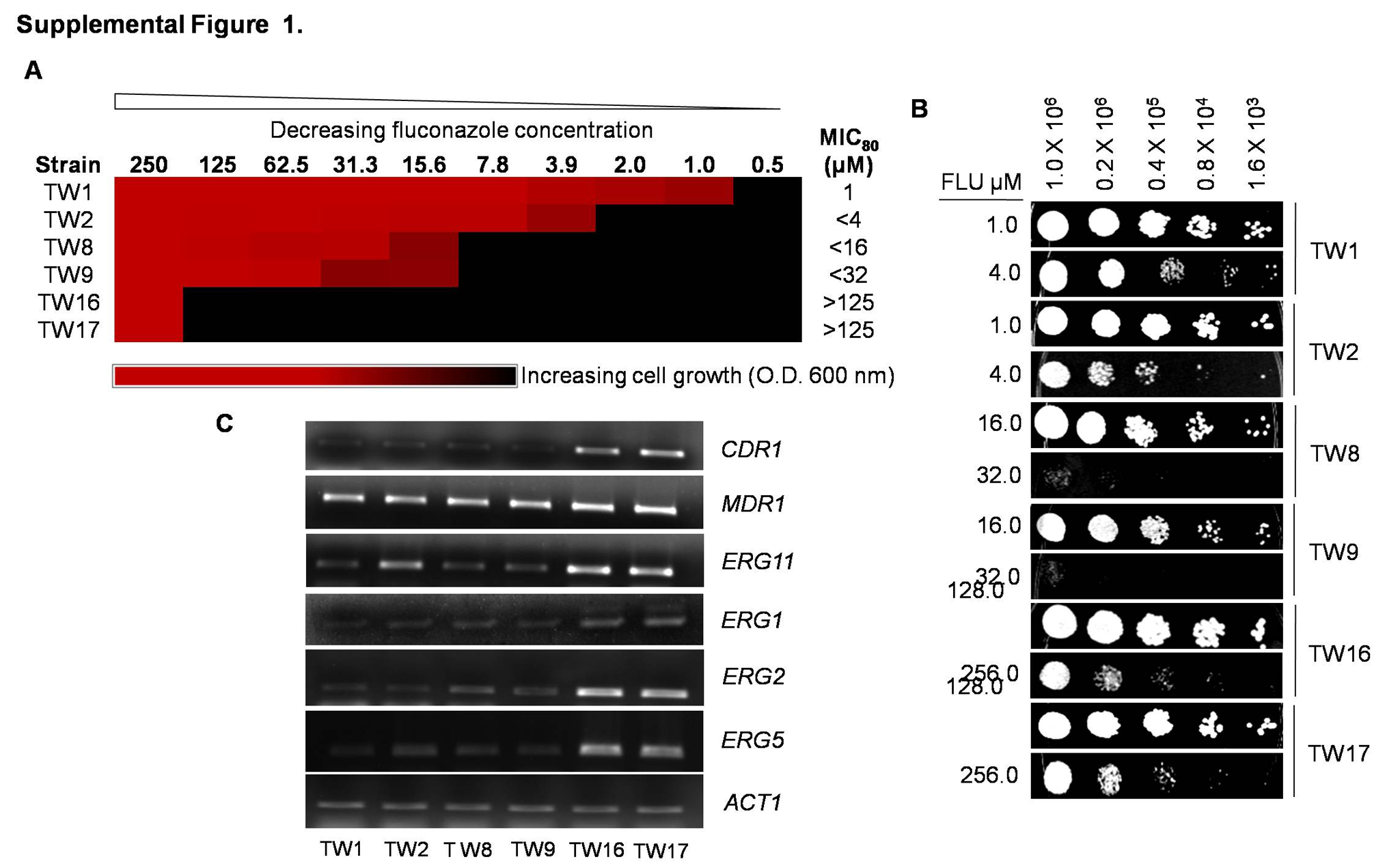

Supplement: Figure S1 — Phenotypic and molecular properties of the sequential isolates of C. albicans used in this study. Isolates are shown on the y axis in the order in which they were obtained from the patient. MICs of FLC were determined by (A) liquid FLC susceptibility assay and (B) the spot assay, as described in Methods. Approximate numbers of cells (per ml) are indicated at the top of panel B. MICs are reported as MIC80 (µM). (C) Genetic changes were identified as the gene expression levels of CDR1, MDR1 and ERG genes as determined by RT-PCR, described in Methods. Values shown as MICs are mean of 3 independent analyses. Spots and RT-PCR result was repeatable at least in 2 independent analyses. (TIF) [file pone.0039812.s001.tif]

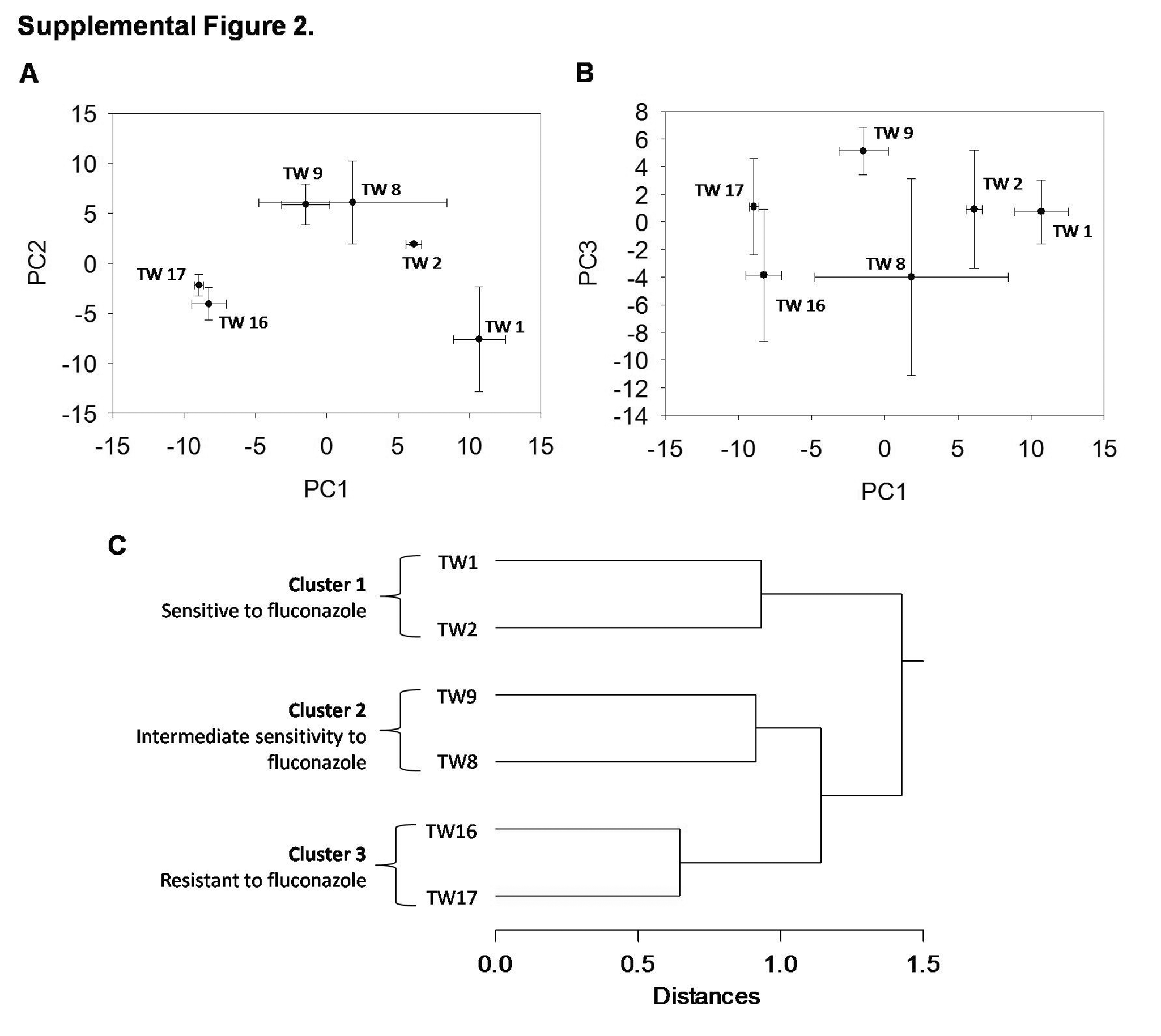

Supplement: Figure S2 — Principal component analysis (PCA) and hierarchical cluster analysis of lipid species amongst the sequential isolates of C. albicans . PCA was performed using the software SYSTAT, version 10 as described in Text S2. The scores for the first three principal components, explaining 53% of the variance, were plotted. Each point in the plot is the mean of corresponding replicate's principal component scores. (A) The scores plot of principal component 1 (27.4% of variance) vs principal component 2 (15.2%). (B) The scores plot of principal component 1 (27.4%) vs principal component 3 (10.8% of variance). (C) Hierarchical cluster dendrogram was prepared using lipidome profiles of the TW isolates. Hierarchical clustering was performed using the software SYSTAT, version 10 as described in Text S2. (TIF) [file pone.0039812.s002.tif]

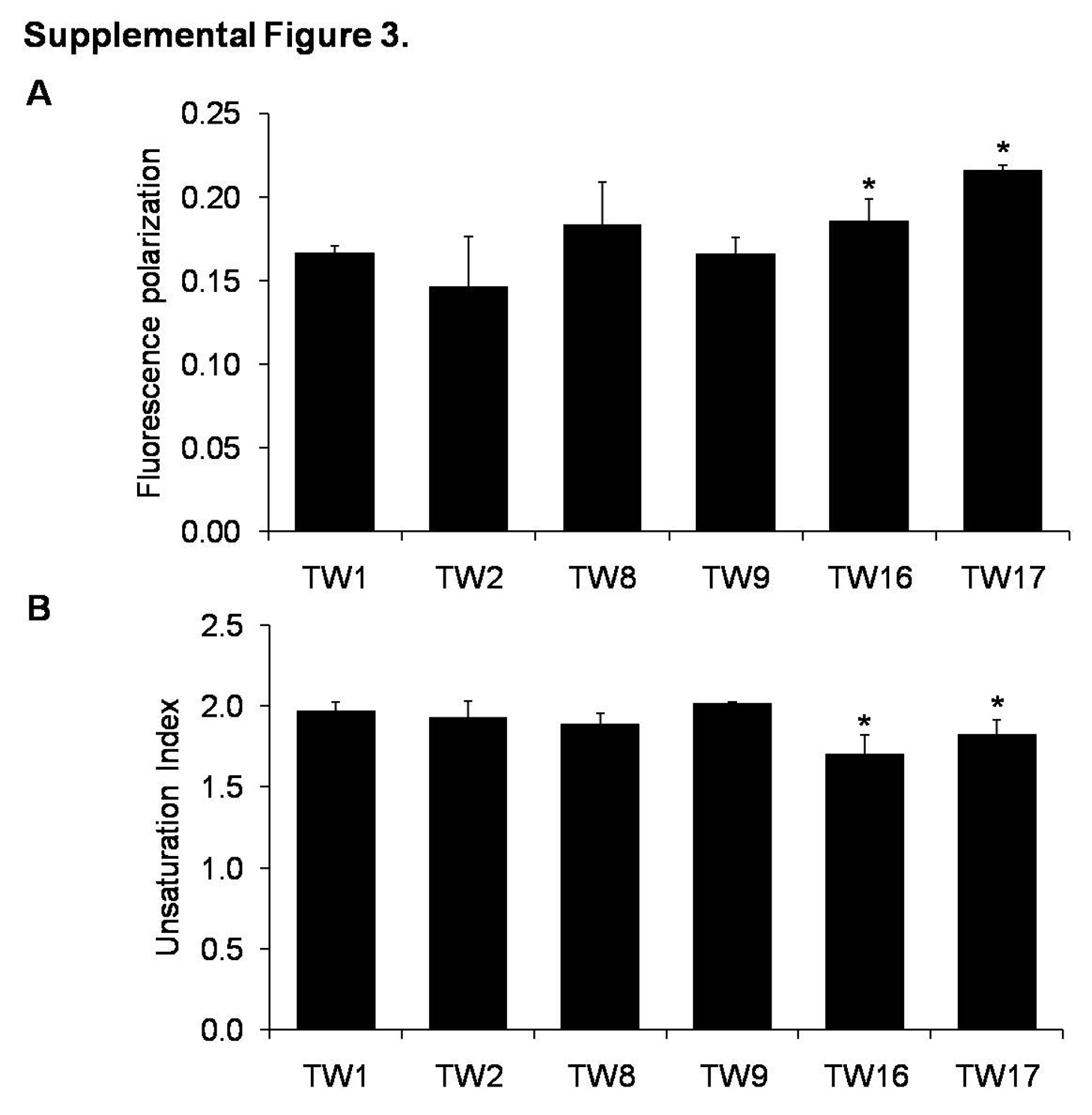

Supplement: Figure S3 — Membrane fluidity and unsaturation index measurements in the sequential isolates of C. albicans . (A) Membrane fluidity was assessed in terms of fluorescence polarization measurements, which were carried out using fluorescent probe 1,6-diphenyl-1,3,5-hexatriene (DPH), as a reporter. Brieﬂy, cells were incubated with Zymolyase (100 U/g wet weight) at 37°C for 3 h with gentle shaking to remove the CW. Spheroplast preparation was monitored turbidometrically by checking the ability of 0.2% sodium dodecyl sulfate to lyse the enzyme-digested cells. Fluorescence polarization was measured at excitation and emission wavelengths of 360 and 426 nm, respectively. The measured ﬂuorescence intensities were corrected for background ﬂuorescence and the light scattering from the unlabeled sample. (B) Degree of unsaturation was determined by calculating the unsaturation index (UI) of the PGLs. UI was calculated as follows: UI = [(1 x % monoene-PGL) + (1 x % diene-PGL) + (1 x % triene-PGL) + (1 x % triene-PGL) + (1 x % tetraene-PGL) + (1 x % pentaene-PGL) + (1 x % hexaene-PGL)]/100. Values are mean of 3 independent analyses (n = 3). Asterisks “*” represents p<0.05. Lipid data taken from Sheet S1, worksheet 3. (TIF) [file pone.0039812.s003.tif]
