# Supplementary material for: Comparative Lipidomics in Clinical Isolates of Candida albicans Reveal Crosstalk between Mitochondria, Cell Wall Integrity and Azole Resistance
Source: PLoS One. 2012 Jun 27;7(6):e39812. doi: 10.1371/journal.pone.0039812 (PMC3384591; doi:10.1371/journal.pone.0039812)

**Table S1. Changes in molecular lipid species upon FLC stress.** Blue and Red colours represent decreasing and increasing trends respectively. The length of the colour in each cell represents the predominance of a lipid species. A total of 242 lipid species were detected, however, table depicts only those lipid species which showed differences between TW1 and TW17 with *p*-values < 0.05. Values are represented as % of the total PGL + SE + SL mass spectral signal and the data taken from Sheet S1, worksheet 3.


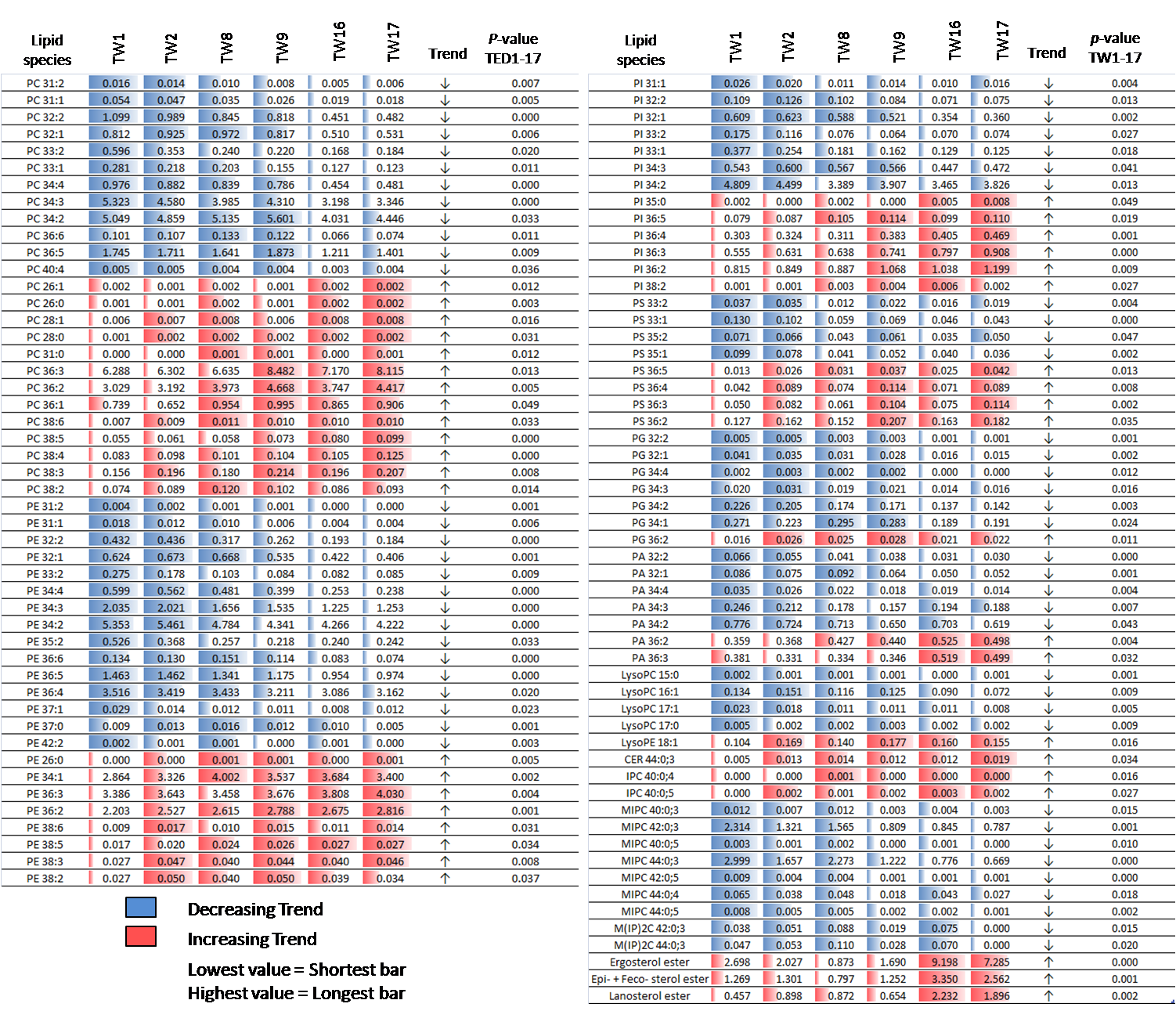

Supplement: Table S1 — Changes in molecular lipid species upon FLC stress. Blue and Red colours represent decreasing and increasing trends respectively. The length of the colour in each cell represents the predominance of a lipid species. A total of 242 lipid species were detected, however, table depicts only those lipid species which showed differences between TW1 and TW17 with p-values <0.05. Values are represented as % of the total PGL + SE + SL mass spectral signal and the data taken from Sheet S1, worksheet 3. (DOC) [file pone.0039812.s006.doc]
