# Supplementary material for: Comparative Lipidomics in Clinical Isolates of Candida albicans Reveal Crosstalk between Mitochondria, Cell Wall Integrity and Azole Resistance
Source: PLoS One. 2012 Jun 27;7(6):e39812. doi: 10.1371/journal.pone.0039812 (PMC3384591; doi:10.1371/journal.pone.0039812)
Supplement: Table S2 — Abundance of PGLs based on their total number of carbons in the FA chains among various isolates used in this study. The data is represented as % of total PGL + SL + SE mass spectral signal after normalization to internal standards. Values are mean of 3 independent analyses (n = 3). Asterisks “*” represents p<0.05. Data taken from Sheet S1, worksheet 3. (DOC) [file pone.0039812.s007.doc]

**Table S2. Abundance of PGLs based on their total number of carbons in the FA chains among various isolates used in this study.** The data is represented as % of total PGL + SL + SE mass spectral signal after normalization to internal standards. Values are mean of 3 independent analyses (n=3). Asterisks “*” represents *p* < 0.05. Data taken from Sheet S1, worksheet 3.

| **No. of carbons** | **TW1** | **TW2** | **TW8** | **TW9** | **TW16** | **TW17** |
| --- | --- | --- | --- | --- | --- | --- |
| **<34-C** | 6.40 ± 0.50 | 5.94 ± 0.33 | 5.18 ± 0.70* | 4.55 ± 0.34* | 3.12 ± 0.06* | 3.23 ± 0.12* |
| **34-C** | 40.28 ± 0.77 | 40.22 ± 2.28 | 40.10 ± 2.27 | 41.38 ± 1.15 | 32.45 ± 2.34* | 34.48 ± 0.41* |
| **36-C** | 32.35 ± 1.19 | 32.62 ± 1.50 | 34.26 ± 2.31 | 38.3 ± 0.25* | 34.2 ± 2.00 | 36.67 ± 1.57* |
| **38-C** | 0.48 ± 0.07 | 0.63 ± 0.05* | 0.62 ± 0.02* | 0.68 ± 0.02* | 0.64 ± 0.09* | 0.70 ± 0.03* |
| **40-C** | 0.03 ± 0.00 | 0.03 ± 0.00 | 0.04 ± 0.00 | 0.04 ± 0.01 | 0.03 ± 0.01 | 0.04 ± 0.01 |
