# Supplementary material for: Comparative Lipidomics in Clinical Isolates of Candida albicans Reveal Crosstalk between Mitochondria, Cell Wall Integrity and Azole Resistance
Source: PLoS One. 2012 Jun 27;7(6):e39812. doi: 10.1371/journal.pone.0039812 (PMC3384591; doi:10.1371/journal.pone.0039812)
Supplement: Table S3 — Changes in the contents of odd chain FA –containing PGL classes among various isolates used in this study. Total amount of odd chain FA-containing PGLs for each lipid class was calculated by adding the normalized amounts of each odd chain FA -containing PGL molecular species of that particular class (namely 31-C, 33-C, 35-C and 37-C containing PGLs). Odd chain FA -containing lipid species were not detected in PA class. The data is represented as % of total PGL + SL + SE mass spectral signal after normalization to internal standards. Values are mean of 3 independent analyses (n = 3). Asterisks “*” represents p<0.05. Data taken from Sheet S1, worksheet 3. (DOC) [file pone.0039812.s008.doc]

**Table S3. Changes in the contents of odd chain FA –containing PGL classes among various isolates used in this study.** Total amount of odd chain FA-containing PGLs for each lipid class was calculated by adding the normalized amounts of each odd chain FA -containing PGL molecular species of that particular class (namely 31-C, 33-C, 35-C and 37-C containing PGLs). Odd chain FA -containing lipid species were not detected in PA class. The data is represented as % of total PGL + SL + SE mass spectral signal after normalization to internal standards. Values are mean of 3 independent analyses (n=3). Asterisks “*” represents *p* < 0.05. Data taken from Sheet S1, worksheet 3.

| **PGL class** | **TW1** | **TW2** | **TW8** | **TW9** | **TW16** | **TW17** |
| --- | --- | --- | --- | --- | --- | --- |
| **PC** | 1.09 ± 0.30 | 0.83 ± 0.03 | 0.71 ± 0.19 | 0.60 ± 0.08* | 0.45 ± 0.03* | 0.48 ± 0.05* |
| **PE** | 1.23 ± 0.46 | 0.92 ± 0.07 | 0.71 ± 0.11 | 0.60 ± 0.06* | 0.62 ± 0.07* | 0.60 ± 0.01* |
| **PI** | 1.36 ± 0.52 | 0.85 ± 0.05 | 0.61 ± 0.15* | 0.6 ± 0.12* | 0.56 ± 0.02* | 0.63 ± 0.05* |
| **PS** | 0.34 ± 0.03 | 0.29 ± 0.04 | 0.16 ± 0.02* | 0.21 ± 0.03* | 0.14 ± 0.02* | 0.15 ± 0.03* |
| **PG** | 0.0003 ± 0.0 | 0.0005 ± 0.0 | 0.001 ± 0.0 | 0.0003 ± 0.0 | 0.0003 ± 0.0 | 0.0004 ± 0.0 |
