# Supplementary material for: Comparative Lipidomics in Clinical Isolates of Candida albicans Reveal Crosstalk between Mitochondria, Cell Wall Integrity and Azole Resistance
Source: PLoS One. 2012 Jun 27;7(6):e39812. doi: 10.1371/journal.pone.0039812 (PMC3384591; doi:10.1371/journal.pone.0039812)
Supplement: Table S4 — Strains used in the study. (DOC) [file pone.0039812.s009.doc]

**Table S4. Strains used in the study.**

| **Strain** | **Reference** |
| --- | --- |
| **TW isolates** | [17] |
| **Gu4** | [67] |
| **Gu5** | [67] |
| **DSY294** | [68] |
| **DSY296** | [68] |
| **DSY544** | [16] |
| **DSY775** | [16] |
| **DSY347** | [16] |
| **DSY289** | [16] |
