# Supplementary material for: Comparative Lipidomics in Clinical Isolates of Candida albicans Reveal Crosstalk between Mitochondria, Cell Wall Integrity and Azole Resistance
Source: PLoS One. 2012 Jun 27;7(6):e39812. doi: 10.1371/journal.pone.0039812 (PMC3384591; doi:10.1371/journal.pone.0039812)
Supplement: Table S5 — Loadings of principal components 1, 2 and 3. The 12 highest and 12 lowest values are indicated. In red colour are shown the molecular lipids species that match with our previous prediction of molecular species that are more responsive during the azole stress [16]. (DOC) [file pone.0039812.s010.doc]

**Table S5. Loadings of principal components 1, 2 and 3.** The 12 highest and 12 lowest values are indicated. In red color are shown the molecular lipids species that match with our previous prediction of molecular species that are more responsive during the azole stress [16].

| **Lipid species 1** | **PC1** | | | **Lipid species 2** | | | **PC2** | | | **Lipid species 3** | **PC3** | | |
| --- | --- | --- | --- | --- | --- | --- | --- | --- | --- | --- | --- | --- | --- |
| *Twelve Lowest loading values* | |  | | |  | | |  | | | |  | |
| PI 36:3 | -0.899 | | | **Ergostatetraenol ester** | | | -0.908 | | | CER 44:0;3 | -0.904 | | |
| PA 36:2 | -0.866 | | | PI 35:2 | | | -0.737 | | | M(IP)_2_C 42:0;3 | -0.804 | | |
| PI 36:2 | -0.820 | | | PE 37:2 | | | -0.703 | | | M(IP)_2_C 44:0;3 | -0.775 | | |
| **Lanosterol ester** | -0.775 | | | PA 36:4 | | | -0.692 | | | IPC 40:0;3 | -0.771 | | |
| PC 38:5 | -0.773 | | | PA 36:5 | | | -0.667 | | | IPC 42:0;3 | -0.720 | | |
| PI 36:4 | -0.773 | | | PA 34:3 | | | -0.606 | | | IPC 44:0;5 | -0.670 | | |
| PE 36:2 | -0.726 | | | PA 36:3 | | | -0.579 | | | IPC 44:0;3 | -0.660 | | |
| **Epi- + Feco- sterol ester** | -0.712 | | | **PE 35:2** | | | -0.575 | | | IPC 44:0;4 | -0.643 | | |
| **Ergosterol ester** | -0.709 | | | PI 33:2 | | | -0.567 | | | **MIPC 42:0;4** | -0.632 | | |
| PA 36:3 | -0.677 | | | **Ergosterol ester** | | | -0.553 | | | **MIPC 40:0;3** | -0.630 | | |
| PE 36:3 | -0.676 | | | PE 33:2 | | | -0.542 | | | IPC 42:0;5 | -0.628 | | |
| PE 38:5 | -0.669 | | | PI 35:1 | | | -0.536 | | | MIPC 44:0;4 | -0.608 | | |
| *Twelve Highest loading values* | | |  | | |  | | |  | | | |  |
| PI 32:1 | 0.885 | | | PE 37:0 | | | 0.641 | | | PG 36:3 | 0.507 | | |
| PE 32:2 | 0.904 | | | PC 36:6 | | | 0.644 | | | PE 36:3 | 0.522 | | |
| **PE 36:5** | 0.905 | | | PI 36:6 | | | 0.666 | | | PS 36:4 | 0.539 | | |
| PC 33:1 | 0.918 | | | PC 34:1 | | | 0.686 | | | PS 34:2 | 0.546 | | |
| PE 31:1 | 0.923 | | | PG 36:2 | | | 0.687 | | | PS 36:1 | 0.558 | | |
| PC 31:2 | 0.923 | | | **PC 30:1** | | | 0.689 | | | PS 36:3 | 0.559 | | |
| PG 34:2 | 0.924 | | | PC 30:2 | | | 0.724 | | | PS 32:2 | 0.566 | | |
| PG 32:1 | 0.937 | | | PS 34:4 | | | 0.733 | | | PC 34:2 | 0.576 | | |
| PC 32:2 | 0.942 | | | PS 34:3 | | | 0.739 | | | PC 36:3 | 0.597 | | |
| PC 34:4 | 0.949 | | | PG 36:4 | | | 0.746 | | | **PC 36:4** | 0.667 | | |
| PC 31:1 | 0.949 | | | PS 34:1 | | | 0.779 | | | **PS 35:2** | 0.677 | | |
| PE 34:4 | 0.983 | | | PC 38:2 | | | 0.789 | | | PS 36:2 | 0.732 | | |
