# Supplementary material for: Comparative Lipidomics in Clinical Isolates of Candida albicans Reveal Crosstalk between Mitochondria, Cell Wall Integrity and Azole Resistance
Source: PLoS One. 2012 Jun 27;7(6):e39812. doi: 10.1371/journal.pone.0039812 (PMC3384591; doi:10.1371/journal.pone.0039812)
Supplement: Text S1 — ESI-MS/MS lipid profiling. (DOC) [file pone.0039812.s011.doc]

**Text S1. ESI-MS/MS lipid profiling.**

***Phosphoglyceride Quantification***:

An automated ESI-MS/MS approach was used. Data acquisition and analysis was carried out as described previously by Devaiah *et al.* and Singh *et al.* [16,21,63] with minor modifications. The extracted dry lipid samples were dissolved in 1 ml chloroform. An aliquot of 2 to 8 µl of extract in chloroform was analyzed, with the exact amount depending upon the dry lipid weight of each sample. Precise amounts of internal standards, obtained and quantified as previously described by Welt*i et al.* and Singh *et al*. [16,26,64], were added in the following quantities (with some small variation in amounts in different batches of internal standards): 0.6 nmol di12:0-PC, 0.6 nmol di24:1-PC, 0.6 nmol 13:0-LysoPC, 0.6 nmol 19:0-LysoPC, 0.3 nmol di12:0-PE, 0.3 nmol di23:0-PE, 0.3 nmol 14:0-LysoPE, 0.3 nmol 18:0-LysoPE, 0.3 nmol di14:0-PG, 0.3 nmol di20:0(phytanoyl)-PG, 0.3 nmol 14:0-LysoPG, 0.3 nmol 18:0-LysoPG, 0.3 nmol di14:0-PA, 0.3 nmol di20:0(phytanoyl)-PA, 0.2 nmol di14:0-PS, 0.2 nmol di20:0(phytanoyl)-PS, 0.23 nmol 16:0-18:0-PI, 0.16 nmol di18:0-PI. The sample and internal standard mixture was combined with solvents, such that the ratio of chloroform/methanol/300 mM ammonium acetate in water was 300/665/35 (v/v/v) in a final volume of 1.4 ml. Each PGL class was quantified in comparison to the two internal standards of that class.

Unfractionated lipid extracts were directly introduced by continuous infusion into the ESI source on a triple quadrupole MS (API 4000, Applied Biosystems, Foster City, CA). Samples were introduced using an autosampler (LC Mini PAL, CTC Analytics AG, Zwingen, Switzerland) fitted with the required injection loop for the acquisition time, and passed to the ESI needle at 30 μl/min.

Sequential precursor (Pre) and neutral loss (NL) scans of the extracts produce a series of spectra revealing a set of lipid species containing a common head group fragment. Lipid species were detected with the following scans: PC and LysoPC, [M + H]^+^ ions in positive ion mode with Pre 184.1; PE and LysoPE, [M + H]^+^ ions in positive ion mode with NL 141.0; PA, [M + NH_4_]^+^ in positive ion mode with NL 115.0; PG, [M + NH_4_]^+^ in positive ion mode with NL 189.0 for PG; PI, [M + NH_4_]^+^ in positive ion mode with NL 277.0; PS, [M + H]^+^ in positive ion mode with NL 185.0; LysoPG, [M – H]^-^ in negative mode with Pre 152.9. The collision gas pressure was set at 2 (arbitrary units (au)). The collision energies, with nitrogen in the collision cell, were +40 V for PC, +28 V for PE, +25 V for PA, +22 V for PG, PI and PS, and –57 V for LysoPG. Declustering potentials were +100 V for PC, PE, PA, PG, PI, and PS, and –100 V for LysoPG. Entrance potentials were +14 V for PC, PA, PG, PI, and PS, +15 V for PE, and –10 V for LysoPG. Exit potentials were +14 V for PC, PA, PG, PI, and PS, +11 V for PE, and –14 V for LysoPG. The mass analyzers were adjusted to a resolution of 0.7 u full width at half height. For each spectrum, 9 to 150 continuum scans were averaged in multiple channel analyzer (MCA) mode. The source temperature (heated nebulizer) was 100°C, the interface heater was “on”, and +5.5 kV or -4.5 kV were applied to the electrospray capillary. The curtain gas was set at 20 au, and the two ion source gases were set at 45 au.

Processing of the data, including isotope deconvolution, was done similar to the way described by Singh *et al.* [16,21]. The background of each spectrum was subtracted, the data were smoothed, and peak areas were integrated using a custom script and Applied Biosystems Analyst software. The lipids in each class were quantified in comparison to the two internal standards of that class. The first and typically every 11^th^ set of mass spectra were acquired on the internal standard mixture only. Peaks corresponding to the target lipids in these spectra were identified and molar amounts were calculated in comparison to the internal standards on the same lipid class. To correct for chemical or instrumental noise in the samples, the molar amount of each lipid metabolite detected in the “internal standards only” spectra was subtracted from the molar amount of each metabolite calculated in each set of sample spectra. The data from each “internal standards only” set of spectra were used to correct the data from the following 10 samples. The analyzed data (in nmol) were normalized to the sample’s “dry lipid weight” to produce data in the units nmol/mg dry lipid weight. Finally, the data were expressed as mole percent of total lipids analyzed.

***Sphingolipid Quantification*:**

The ESI-MS/MS procedure for SL quantification was performed as described previously by Singh *et al.* [16,21]. Lipid species were detected with the following scans: CER, [M + H – H_2_O]^+^ ions in negative ion mode with Pre 300 and Pre 328; IPC, [M - H]^-^ ions in negative ion mode with Pre 259; MIPC, [M - H]- ions in negative ion mode with Pre 421; M(IP)2C, [M - H]^-^ in negative mode with Pre 663.1. The internal standard, 16:0-18:1-PI, was detected with [M - H]^-^ in negative ion mode with Pre 241.0, as described in the previous section. The CER internal standard, t18:0-phytoceramide, was detected with [M + H – H_2_O]^+^ in positive ion mode with Pre 300. The collision gas pressure was set at 2 au. The collision energies, with nitrogen in the collision cell, were +43 V for CER, -72 V for IPC, -80 V for MIPC, and -75 V for M(IP)_2_C. The declustering potential was +180 V for CER and -180 V for IPC, MIPC, and M(IP)_2_C. The exit potential was +10 V for CER and -15 V for IPC, MIPC, and M(IP)_2_C. The mass analyzers were adjusted to a resolution of 0.7 u full width at half height. For each spectrum, 125 to 250 continuum scans were averaged in multiple channel analyzer (MCA) mode. The source temperature (heated nebulizer) was 100°C, the interface heater was “on”, and +5.5 kV or -4.5 kV were applied to the electrospray capillary. The curtain gas was set at 20 au, and the two ion source gases were set at 45 au. The rest of the processing was similar to that for phosphoglycerides; however, all SL signals were normalized to the signal for 0.23 nmol 16:0-18:0-PI that was added in as an internal standard.

SL amounts were determined by normalizing the mass spectral signal so that a signal of 1.0 represents a signal equal to the signal of 1 nmol 16:0-18:0-PI (the internal standard). These data were then divided by the sample dry weight to obtain signal/dry weight, by the total SL signal to obtain % of total SL signal. It is possible that there is variation in ionization efficiency among various SLs and between the internal standard and SL species. Thus, normalized SL species amounts may not reflect their molar amounts. However, the employed procedure allows for determination of relative abundance of SLs and comparison of amounts of particular SLs among samples.

*Sterol esters quantification:*

The ESI-MS/MS procedure for SE quantification was was performed as described previously by Singh *et al.* [16]. Precise amounts of internal standards were added in the following quantities (with small variations of amounts in different batches of internal standards): 4.6 nmol di15:0-DAG. Lipid species for SE were detected with the following scans: 16:1, 16:0, 18:3, 18:2, 18:1 and 18:0 -containing species, were detected as [M + NH_4_]^+^ in positive ion mode with NL 271.2, 273.2, 295.2, 297.2, 299.2 and 301.2 respectively. The internal standard di15:0-DAG was detected with [M + NH_4_]^+^ in positive ion mode with NL 259.2. The collision gas pressure was set at 2 au. The collision energy, with nitrogen in the collision cell, was +25 V for SE. The declustering potential was +100 V for SE. The exit potential was +12 V for SE. The mass analyzers were adjusted to a resolution of 0.7 u full width at half height. For each spectrum, 50 continuum scans were averaged in multiple channel analyzer (MCA) mode. The source temperature (heated nebulizer) was 100°C, the interface heater was “on”, and +5.5 kV were applied to the electrospray capillary. The curtain gas was set at 20 au, and the two ion source gases were set at 45 au. The rest of the processing was similar to that for PGLs; however, all SE signals were normalized to the signal for 4.6 nmol di15:0-DAG that was added in as an internal standard.

For these samples, SE are represented as a list of "fatty acid containing" species. So, there are the 16:0 containing species, the 18:1 containing species and so on. SE amounts were determined by normalizing the mass spectral signal so that a signal of 1.0 represents a signal equal to the signal of 1 nmol di15:0-DAG (the internal standard). The SE data were then divided by the dry lipid weight to obtain signal/dry lipid weight, by the total SE signal to obtain % of total SE signal. It is possible that there is variation in ionization efficiency among various SEs and between the internal standard and SE species. Thus, normalized SE species amounts may not reflect their molar amounts. However, the employed procedure allows for determination of relative abundance of SEs and comparison of amounts of particular SEs among samples. Of note, the total amount of each SE class has been calculated by adding the normalized mass spectral signal of all respective FA containing SE (for example, total amount of ergosterol esters was calculated by adding the mass spectral signal of 16:1, 16:0, 18:3, 18:2, 18:1 and 18:0 –containing ergosterol ester).
