# Supplementary material for: Comparative Lipidomics in Clinical Isolates of Candida albicans Reveal Crosstalk between Mitochondria, Cell Wall Integrity and Azole Resistance
Source: PLoS One. 2012 Jun 27;7(6):e39812. doi: 10.1371/journal.pone.0039812 (PMC3384591; doi:10.1371/journal.pone.0039812)
Supplement: Text S2 — Principal Component Analysis (PCA) and Hierarchal cluster analysis of lipid species quantitative data distinctly separates susceptible and resistant isolates. (DOC) [file pone.0039812.s012.doc]

**Text S2. Principal Component Analysis (PCA) and Hierarchal cluster analysis of lipid species quantitative data distinctly separates susceptible and resistant isolates.**

PCA plots allow visual assessment of the similarities and differences among samples and help to determine whether and how samples can be grouped [16,21,63]. To confirm if the observed changes in molecular species of lipids represent statistically significant variations between the TW isolates, the principal component analysis (PCA) was performed using the molecular species percentage composition of PGL + SL + SE (Sheet S1, worksheet 3).

As shown in the plot of principal component 1 versus 2 (Figure S2A), principal component 1 describes the separation of TW1 from the other isolates of this series, particularly from TW 8 and 9 (intermediate susceptibility to FLC) and TW16 and 17 (resistant to FLC). The highest and lowest loading values reflect the lipid species that are most important in the assignment of each principal component (Table S5). Examination of the loadings associated with principal component 1 (Table S5) shows that the content of 36 and 38 carbon PGLs with two or more double bonds (PC 38:5, PEs 36:2, 36:3, and 38:5, PAs 36:2 and 36:3, and PI 36:2, 36:3 and 36:4) are important for the separation of TW isolates along the negative principal component 1 axis, while the content of 31 to 34 carbon PGLs with one or two double bonds (PI 32:1, PEs 31:1 and 32:2, PCs 31:1, 31:2, 33:1 and 32:2, and PGs 32:1 and 34:2) along with SEs are important for the separation of TW isolates along the positive principal component 1 axis. Interestingly, the amounts of all molecular lipid species associated with the lowest and highest loading values of principal component 1 axis showed an increasing and decreasing trend respectively, from TW1 to TW17 (viz. increasing FLC concentration). However, the amounts of SEs associated with the lowest loading values of principal component 1 also showed an increasing trend with some depletion in TW8 and TW9.

Principal component 2 differentiates several TW isolates (Figure S2A); for example it describes the variation between TW1 and TW2 as compared to TW8 and TW9, and TW16 and TW17. The lowest loadings for principal component 2 point to decreasing trends in amounts of PE 33:2, 35:2 and 37:2, PI 33:2, 35:1 and 35:2, upon increasing FLC resistance with highest amounts observed in TW1. PE 33:2, PI 35:1 and 35:2 amounts were slightly higher in TW17 compared to TW16. PE 35:2 amounts were slightly higher in TW9. Decreasing trends in amounts of PA 34:3, 36:4 and 36:5, upon increasing FLC resistance were observed from TW1 to TW8 or 9, but the highest amounts of these molecular species was observed in TW16 and TW17. The amounts of PA 36:3 and few SEs (namely ergostatetraenol and ergosterol esters) showed an increasing trend, but ergostatetraenol esters were higher in TW1 compared to TW2 to TW9 and ergosterol esters were partially depleted in TW8 and 9. The highest loadings for principal component 2 point to increasing trends in amounts of PC 30:1, 30:2, 36:6 and 38:2, PE 37:0, PI 36:6, PS 34:1, 34:3 and 34:4 from TW 1 to TW8 or 9, while the amounts of these molecular species show a decreasing trend from TW9 or 16 to TW17, with maximum amounts of these species in TW8 or TW9. The amounts of PG 36:2 and 36:4 shows an increasing trend with highest amounts of these species in TW2 and TW8 respectively. Therefore, loadings of principal component 2 effectively separate TW 8 and 9 from TW1 and 2, and TW16 and 17 (Figure S2A).

Principal component 3 describes the difference between TW8 and TW9 (Figure S2B). The loading values for principal component 3 indicate that the separation between the two isolates reflect higher levels of several PSs (32:2, 34:2, 35:2, 36:1, 36:2, 36:3 and 36:4) and PCs (34:2, 36:3 and 36:4) in TW9 and many SL species (CER 42:0;3, IPC 40:0;3, 40:0;4 and 44:0;3, MIPC 40:0;3, M(IP)_2_C 42:0;3 and 44:0;3) in TW8. Lower amounts of IPC 44:0;4, MIPC 44:0;3 and 44:0;4 species in TW9.

Overall, PCA could validate statistically significant variations in the molecular lipid species of TW isolates. Also, PCA clearly demarcates TW1/TW2, TW8/TW9 and TW16/TW17 into 3 different clusters based on their molecular lipid imprints. Further PCA highlights the molecular species that mark the transition from TW1→ TW2 ----→ TW8 → TW9 -----→TW16 →TW17.

Hierarchal clustering provides valuable information regarding the degree of closeness among the datasets [21]. We have clustered lipid profiles of various *Candida* isolates used in this study as described in methods. Based upon their lipid profiles, TW isolates could be clustered into 3 groups (Figure S2C). TW1 and TW2 were part of one cluster and were sensitive to FLC (Figure S2C and Figure S1). TW8 and TW9 were part of second cluster and were moderately resistant to FLC (Figure S2C and Figure S1). TW16 and TW17 were part of the third cluster and were most resistant to FLC (Figure S2C and Figure S1). Therefore, this hierarchal clustering re-confirms our PCA results that a certain set of molecular species are responsible for the segregation of one cluster from the other.
